# No evidence of PEDV infection in French artificial insemination centers in 2015

**DOI:** 10.1186/s40813-018-0110-9

**Published:** 2019-01-15

**Authors:** S. Gallien, V. Catinot, N. Pozzi, M. Berri, E. Authié, N. Rose, B. Grasland

**Affiliations:** 1 ANSES, Agence Nationale de Sécurité Sanitaire, Laboratoire de Ploufragan, B.P.53, 22440 Ploufragan, France; 2 Université Bretagne Loire, 35000 Rennes, France; 3 ISP, INRA, Université François Rabelais de Tours, UMR 1282, 37380 Nouzilly, France; 4 Laboratoire National de Contrôle des Reproducteurs (LNCR), 94700 Maisons-Alfort, France

**Keywords:** Porcine epidemic diarrhea virus, Boars, French artificial insemination centers, Serology

## Abstract

Pigs infected by porcine epidemic diarrhea virus (PEDV) are affected by severe diarrhea, vomiting and dehydration. The severity of clinical signs depends on the virus strain. Two genetically different PEDV strains are known to infect pigs, the PEDV S-InDel strains which circulate on all continents and the highly virulent PEDV S-non-InDel strains found in Asia and in America. We have previously demonstrated the presence of PEDV RNA in semen from boars experimentally infected with an S-non-InDel PEDV strain. If naturally infected boars may shed PEDV in semen, this would have important consequences for the breeding sector. Thus we sought to determine whether PEDV has been circulating in populations of breeding boars from French artificial insemination (AI) centers. The current study reports on a serological survey conducted on one hundred and twenty boars from six AI centers, representing 18.6% of the total population of breeding boars in French AI centers in 2015. All of them were found negative for PEDV antibodies, showing no evidence of PEDV circulation in French AI centers at that time.

The first case of porcine epidemic diarrhea (PED) was reported in the 1970s in Europe. The disease is caused by an enveloped positive single-stranded RNA *alphacoronavirus* called porcine epidemic diarrhea virus (PEDV). Clinically, the disease is characterized by a severe profuse diarrhea with or without vomiting and dehydration, resulting in high morbidity and mortality, particularly in suckling piglets’ populations. The economic losses due to PED can thus be very important. Nowadays, PEDV circulates on the Asian, American and European continents [1]. Different strains have been described in these continents which can be clustered according to the nucleotide sequence of the S gene. Highly virulent strains named PEDV S-non-InDel strains have been reported on the Asian and American continents since 2010 and 2013, respectively [1–3]. In North America, those strains were responsible for large epidemics and are currently endemic in the US pig population.

So far, only one case of PEDV S-non-InDel strain has been reported in Europe, in Ukraine, in 2014 [4]. The PEDV S-InDel strains which are responsible of less severe clinical cases than the PEDV S-non-InDel strains circulate on all three continents [2, 3, 5]. The major route of transmission of this virus is the fecal-oral route through direct and indirect contacts with infected pigs or contaminated feces. Staff from infected farms, as well as contaminated equipment, including trailers can carry the virus. Airborne transmission is also possible [1]. Finally the presence of PEDV genome in semen has been shown in boars from boar studs in the US and Canada [6] and we have demonstrated that the genome of a PEDV S-non-Indel strain could be shed for several days in semen by experimentally infected boars after oral inoculation [7]. Worldwide, the commercial trade of breeding boars and semen is important and the risk of propagation of infectious diseases by this route has to be considered [8]. If infectious PEDV may be shed in semen from naturally infected boars, this would have important consequences for the breeding sector.

Thus we sought to determine if PEDV has been circulating in breeding boars from French artificial insemination (AI) centers. To this aim we conducted a serological survey on 120 boars from 6 AI centers, representing 18.6% of the total population of breeding boars in French AI centers in 2015. Four of the 6 AI centers were located in Brittany where the pig production is the most densely populated. The two remaining AI centers were located near the border with countries where several PED cases were declared at the end of 2014 and in 2015 (Fig. 1). Blood samples were collected every four months from 48 boars, every three months from 69 boars, twice a year for two boars and once a year for one boar. Blood samples were centrifuged at 12000×*g* for 10 min and the sera were stored at − 20 °C. Sera were tested for PEDV N antibodies using a commercial ELISA test, ID Screen® PEDV Indirect (ID Vet, Grabels, France) based on the N protein. According to the manufacturer’s recommendation, the ELISA test is considered valid if the mean OD (optical density) value obtained with the positive control is greater than 0.350 and if the ratio of the mean values given by the positive and negative controls is greater than 3. For each sample to test, the S/P (sample-to-positive) ratio was calculated. Samples with S/P ratio equal or higher than 60% were considered positive for PEDV antibodies. The specificity and the sensitivity of this ELISA test are 0.99 and 0.8 respectively [9].

**Fig. 1:**
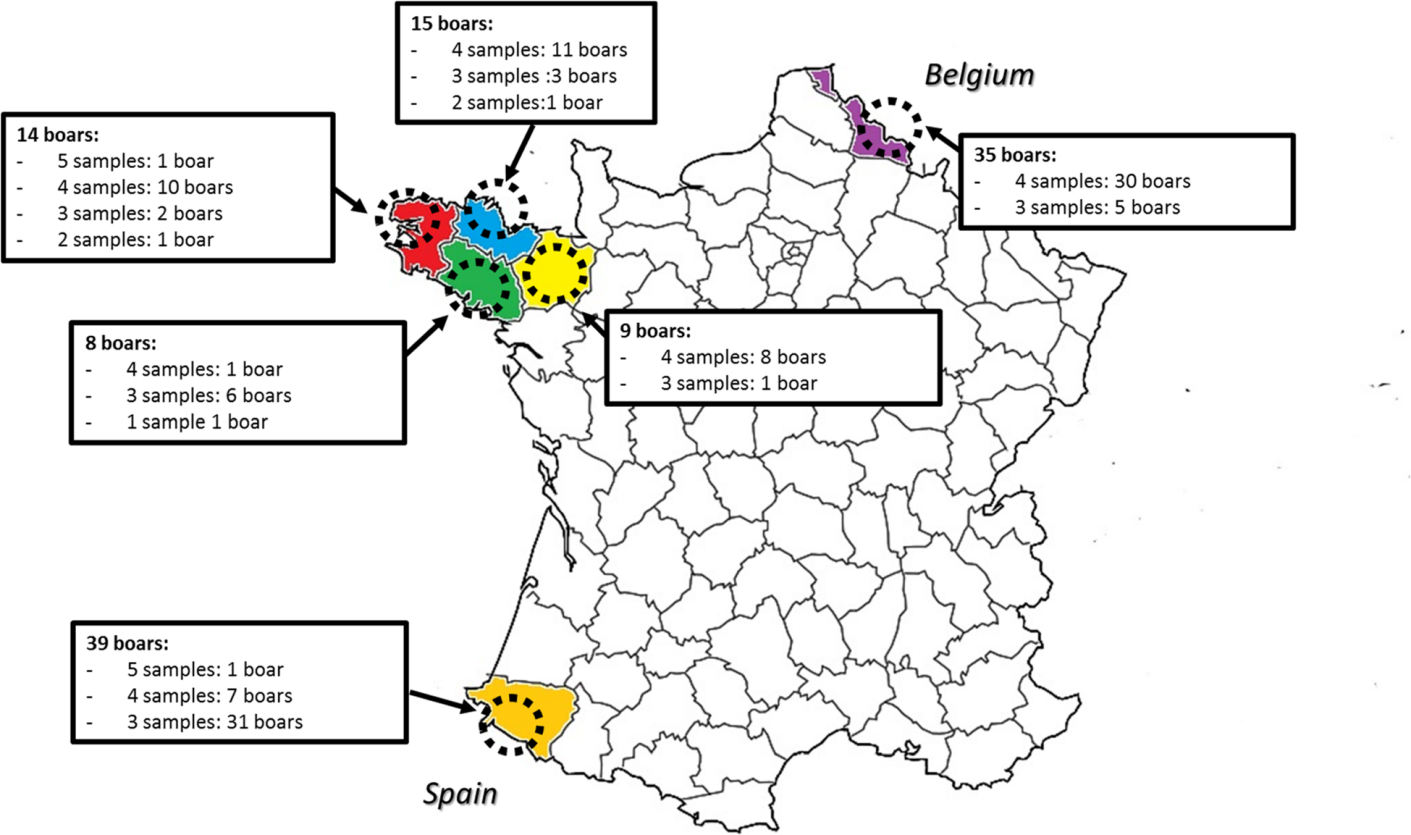
Repartition of boars sampled and number of blood samples for each boar in France during the year 2015

All the ELISA tests performed were validated using manufacturer criteria. None of the tested boars were found positive for PEDV antibodies with a 95% confidence interval ranging from 0 to 1.4% (accounting for the sample size and the sampling fraction from the whole population). Boars did not show clinical signs of the PED between each samples points. These results suggest that, as the end of 2015, PEDV has not been circulating in the populations of breeding pigs from the French IA centers tested in this study.

Breeding boars and porcine semen are currently imported from various countries in the world to improve pig genetics. There may be importations from countries where PEDV non-InDel and/or PEDV InDel strains have been circulating and thus the risk of spreading this pathogen has to be considered. In France, the marketed semen are not subjected to systematic testing for the presence of PEDV, but the import of boars from countries where PEDV non-InDel strains are circulating is regulated. A systematic control of boars introduced from other countries has been recently implemented by the French pig breeding sector. Prior to movements, a veterinary certificate has to be issued for each boar, attesting the absence, during the previous year, of clinical signs of PED, seroconversion and virus detection in the farm from which the boars are originating. Upon arrival in France, imported boars are quarantined for a period of 30 days. A second quarantine of 30 days may be applied before entry into semen collection centers. In semen collection centers boars are routinely tested for Classical Swine Fever, Brucellosis and Aujeszky’s disease as a regulatory requirement [10]. In addition, although it is not mandatory, a diagnostic test for PEDV (ELISA test or PEDV specific RT-qPCR) is most of the time performed at the breeding sector’s initiative.

Our results indicate that the sanitary controls of boars prior their departure from the holding of origin and before their entry into semen collection centers may have prevented PEDV introduction into French AI centers till 2015. Nevertheless, there remain some uncertainties on the PEDV status of imported boars. A recent study revealed that clinically healthy boars experimentally infected with PEDV (absence of diarrhea, vomiting and shedding in feces) could shed intermittently PEDV in semen in the absence of seroconversion [7]. The veterinary certificate delivered prior to departure of the breeding boars might therefore not provide absolute certitude of safe semen. It might be advisable to carry out PCR on semen samples for artificial insemination.

To our knowledge, there is no report from other countries on the serological status of boars in AI centers. Our study indicates that PEDV has most likely not circulated in the French AI centers included in the study, at least till 2015. Nevertheless it might be advisable, in order to ensure safe trade and safe AI, to recommend semen testing for PEDV. AI centers in France have adopted for a long time good practices in routine work and thus have reached a high level of biosecurity. It is essential that this is maintained and possibly reinforced in order to prevent introduction of emerging pathogens such as PEDV.

## Abbreviations

AI: Artificial Insemination
ELISA: Enzyme-linked immunosorbent assay
OD: Optical Density
PCR: Polymerase chain reaction
PED: Porcine Epidemic Diarrhea
PEDV: Porcine Epidemic Diarrhea Virus
